# Drug-Resistant Cholangiocarcinoma Cell Lines for Therapeutic Evaluation of Novel Drugs

**DOI:** 10.3390/molecules30143053

**Published:** 2025-07-21

**Authors:** Kevin Delgado-Calvo, Elisa Lozano, Oscar Briz, Candela Cives-Losada, Jose J. G. Marin, Rocio I. R. Macias

**Affiliations:** 1 Experimental Hepatology and Drug Targeting (HEVEPHARM) Group, Institute of Biomedical Research of Salamanca (IBSAL), Center for the Study of Liver and Gastrointestinal Diseases (CIBERehd), University of Salamanca, Campus M. Unamuno s/n, 37007 Salamanca, Spain; kevindc@usal.es (K.D.-C.); obriz@usal.es (O.B.); candela.cives_losada@uniupo.it (C.C.-L.); jjgmarin@usal.es (J.J.G.M.); 2 Department of Health Sciences, University of Piemonte Orientale, 28100 Novara, Italy; 3 Hepatobiliary Immunopathology Laboratory, IRCCS Humanitas Research Hospital, Rozzano, 20089 Milan, Italy

**Keywords:** 5-fluorouracil, biliary cancer, chemoresistance, cisplatin, multidrug resistance, preclinical model

## Abstract

The pharmacological treatment of cholangiocarcinoma (CCA) is often hampered by tumor resistance. Improving our understanding of this issue is crucial for developing strategies that can overcome drug refractoriness. We have established and characterized two novel human cell sublines derived from extrahepatic CCA EGI-1 cells that are resistant to cisplatin and 5-fluorouracil (5-FU). Migration and proliferation were analyzed using holographic microscopy. The expression of genes involved in drug uptake and efflux was determined by RT-qPCR. Cross-resistance to commonly used antitumor drugs was assayed using the MTT test. EGI-1 sublines resistant to cisplatin (CR) or 5-FU (FR) exhibited more than a three-fold increase in resistance to cisplatin and 5-FU, respectively, and showed reduced proliferation, migration, and colony-formation rates, along with an altered cell cycle compared to wild-type cells, while retaining tumorigenic capacity. The analysis of the transportome showed downregulation of uptake transporters and upregulation of the export pumps MRP3/4. EGI-1 cells with acquired resistance to 5-FU demonstrated cross-resistance to irinotecan and gemcitabine, while cisplatin-resistant cells showed decreased sensitivity to 5-FU and platinum derivatives. These resistant cell lines offer valuable models for investigating the molecular basis of chemoresistance in CCA, providing a robust platform for the development and evaluation of novel therapeutic strategies.

## 1. Introduction

Cholangiocarcinoma (CCA) is a highly heterogeneous and aggressive tumor that originates from bile duct epithelial cells [1]. Based on their anatomical site of origin, CCAs are categorized into intrahepatic (iCCA), perihilar (pCCA), and distal (dCCA) subtypes. Worldwide incidence and mortality rates of this biliary cancer have shown a steady upward trend over the last 30 years and are believed to be underestimated in Western countries [2]. In Europe, the average CCA-related mortality is >2 per 100,000 population, with considerable variability between countries, partly due to disparities in the access to diagnostic imaging, molecular profiling, and novel therapeutics across European countries [3].

Most CCAs are diagnosed in patients with no risk factors and at an advanced stage because early disease is asymptomatic or presents with nonspecific symptoms. Due to late diagnosis, many patients are not candidates for surgical intervention (the only curative option), and their prognosis is dismal. Even though the number of targeted therapy drugs available has considerably increased in recent years [4,5,6,7], the first-line treatment of locally advanced or metastatic CCA consists of the combination of cisplatin/gemcitabine with immune checkpoint inhibitors durvalumab or pembrolizumab, after demonstrating moderate improvement in overall survival in clinical trials [8,9]. The combination of 5-fluorouracil (5-FU), leucovorin, and oxaliplatin (FOLFOX) is the preferred second-line treatment, although its impact on survival is also modest [10]. Capecitabine is commonly administered after surgery [11,12]. Other agents, such as paclitaxel or irinotecan, have been explored in combination therapies and are used in subsequent treatment lines, but none have demonstrated superior efficacy.

The fact that these tumors are resistant to pharmacological treatments has a severe impact on their clinical efficacy, and it is also a major contributor to patients’ poor prognosis [1]. A comprehensive understanding of the molecular mechanisms that drive chemoresistance is essential for advancing towards personalized treatment strategies capable of overcoming resistance. Significant efforts are underway to identify new therapeutic targets and synthesize more effective drugs for CCA. For example, inhibiting the enzyme PRMT5 has yielded promising results by stopping tumor growth [13]. The development of cytostatic bile acid derivatives represents an innovative approach to enhance treatment efficacy. Some of these compounds, known as Bamets, are conjugates of bile acids and cisplatin designed to maintain the antitumor activity of the cytostatic agent and increase drug accumulation in hepatobiliary tumor cells while reducing systemic toxicity. Preclinical studies demonstrated that Bamets, such as Bamet-UD2 and Bamet-R2, exhibit significant antiproliferative effects by interfering with cancer cell metabolism and inducing apoptosis, making them promising candidates for the treatment of hepatobiliary tumors [14,15,16].

Given the challenges posed by such a complex, heterogeneous, and aggressive disease, models such as drug-resistant cell lines are valuable tools for advancing our understanding of mechanisms of chemoresistance (MOCs). Moreover, these models can help identify new potential therapeutic targets, test novel agents, and develop novel therapeutic strategies aimed at enhancing treatment response [17,18]; these may include optimized drug combinations, sequential therapies, or personalized treatment adaptations based on tumor-specific changes following drug exposure.

Previous studies have established CCA cell lines resistant to gemcitabine [19,20], which have helped characterize the mechanisms involved in resistance to this antimetabolite. In this study, we aimed to establish and characterize cisplatin- and 5-FU-resistant CCA cell lines to better understand the adaptive responses driving the lack of response to these agents. Most existing CCA-resistant models focus on gemcitabine, making cisplatin/5-FU-resistant lines a novel contribution. Through stepwise drug exposure, we established cell models resistant to cisplatin or 5-FU and conducted comprehensive analyses to identify the molecular changes underlying acquired resistance related to transport proteins involved in drug uptake and efflux. Additionally, we assessed the effectiveness of additional chemotherapeutic agents in these resistant cell lines, identifying drugs that exhibited cross-resistance and others that retained effectiveness.

## 2. Results

### 2.1. Development and Characterization of New Resistant Cholangiocarcinoma Cell Lines

**Figure 1:**
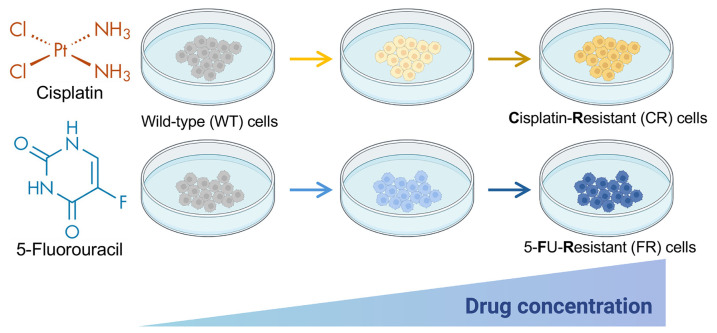
Schematic representation of the procedure for obtaining EGI-1 cholangiocarcinoma cell lines resistant to cisplatin (CR) or 5-fluorouracil (FR) after 9 months of exposure to increasing concentrations of the anticancer agents. The starting concentration was 2 µM for cisplatin and 0.5 µM for 5-flurouracil (5-FU), and the final concentration was 9 µM for cisplatin and 10 µM for 5-flurouracil. Partially created with BioRender.com. Arrows depict successive culture passes with a stepwise increase in drug concentrations.

Resistant cells were generated by continuously exposing EGI-1 parental cells to increasing concentrations of each drug for a period of 9 months, starting at a concentration corresponding to the doses that reduced a 5% cell viability, i.e., 2 µM for cisplatin and 0.5 µM for 5-FU, and going up to 9 µM for cisplatin and 10 µM for 5-FU (Figure 1). The culture medium was changed twice a week, passages were performed approximately every two weeks, and when cells reached around 80% confluence, drug doses were progressively increased every 10–14 days.

**Figure 2:**
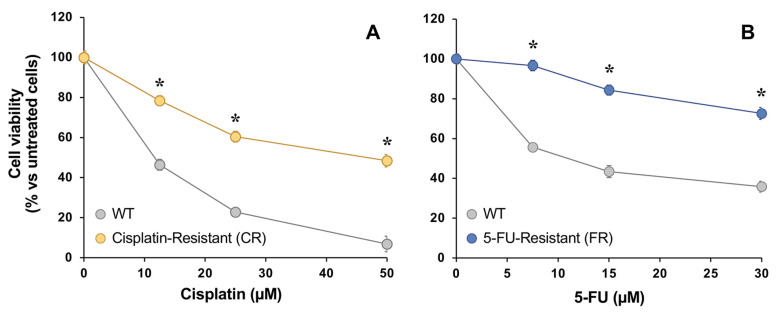
Concentration-dependent effect of cisplatin (**A**) and 5-FU (**B**) on the viability of EGI-1 cells determined with the MTT-formazan test after 72 h exposure. Cells were either wild-type (WT) or resistant to cisplatin (CR) or 5-FU (FR). Values are expressed as the percentage of viability relative to untreated cells and are the mean ± SEM of three separate experiments performed in triplicate. *, *p* < 0.05, comparing each resistant cell line with WT cells.

Viability assays were performed to assess if there were sensitivity changes in EGI-1 cells exposed to increasing concentrations of either cisplatin or 5-FU in comparison with the parental wild-type (WT) cell line (Figure 2). The IC_50_ values of cisplatin in cisplatin-resistant (CR) cells and of 5-FU in 5-FU-resistant (FR) cells increased more than three-fold compared with their parental cells.

**Figure 3:**
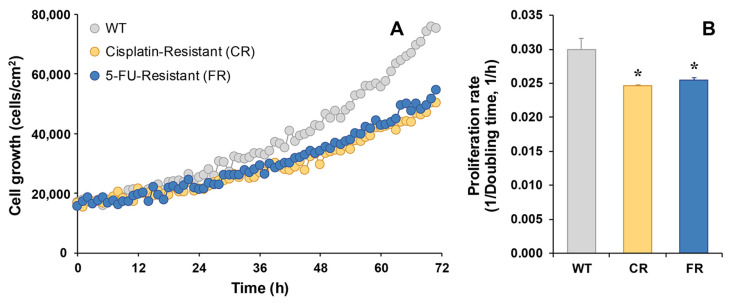
Representative recording of real-time proliferation of EGI-1 wild-type (WT) cells or cells resistant to cisplatin (CR) or 5-FU (FR) as monitored by holographic phase imaging (**A**). Images of 8 regions per well were captured every 60 min for 72 h and analyzed with the appropriate software. The proliferation rate was calculated as the inverse of the doubling time (**B**). Values are the mean ± SEM of three separate experiments performed in duplicate. *, *p* < 0.05, comparing each resistant cell line with WT cells.

Cell proliferation, as determined by holographic microscopy, was slower in the CR and FR than in EGI-1 WT cells (Figure 3A), with a significantly lower proliferation rate in both resistant cell lines (Figure 3B). Specifically, the doubling time of EGI-1 WT cells was 33 h, while FR cells required 39 h and CR cells 41 h to double.

**Figure 4:**
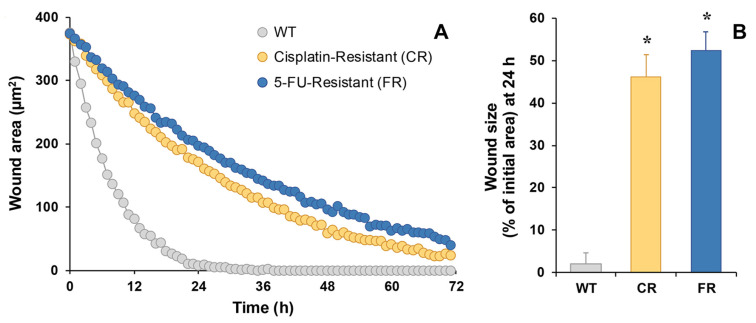
Representative recording of time course of the wound size performed on EGI-1 WT or resistant cells (CR and FR) as monitored by holographic phase imaging (**A**). Images of 8 regions per well were captured every 60 min for 72 h and analyzed with the appropriate software. Wound healing at 24 h expressed as the percentage of the initial wound area (**B**). Values are the mean ± SEM of three separate experiments performed in duplicate. *, *p* < 0.01, comparing each resistant cell line with WT cells.

Cell migration, determined by the wound-healing assay, also detected using holographic microscopy, confirmed that both EGI-1 resistant cells had less migration capacity compared to parental cells, as the observed wound area remained significantly larger (Figure 4A). In fact, the wound was almost closed after 24 h in EGI-1 WT cells, being 2% of the initial size (Figure 4B), while only 46% and 52% were reached in the case of CR and FR cells, respectively.

**Figure 5:**
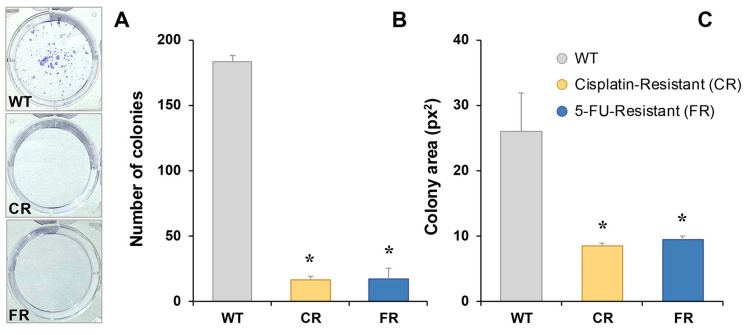
Representative images of colony formation in EGI-1 wild-type (WT), cisplatin-resistant (CR) and 5-fluorouracil-resistant (FR) cells stained with crystal violet 7 days after seeding (**A**). Quantification of colony-forming units (**B**) and mean colony area (**C**). Results represent the mean ± SEM of three independent measurements. *, *p* < 0.05, comparing each resistant cell line with WT cells.

Colony formation was greatly slowed in chemoresistant EGI-1 cells (Figure 5). Both CR and FR cells formed fewer (Figure 5A,B) and smaller (Figure 5A,C) colonies compared to the parental cell line.

**Figure 6:**
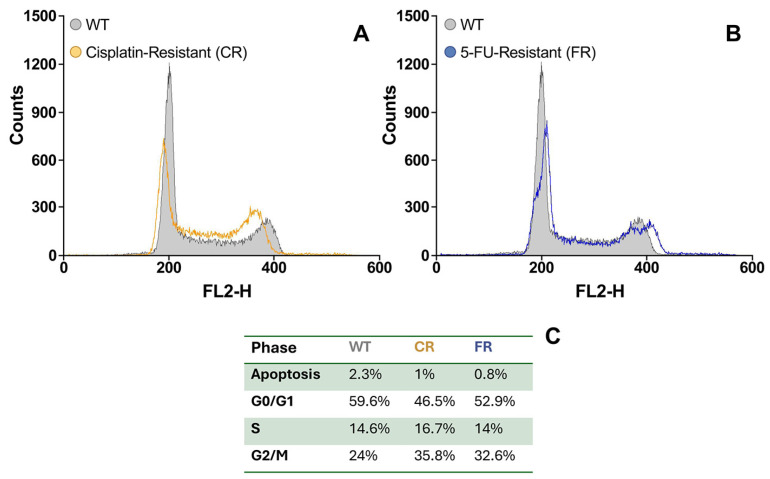
Representative flow cytometry histograms of cell populations in cisplatin-resistant (CR) cells (**A**) and 5-fluorouracil-resistant (FR) cells (**B**) compared to EGI-1 wild-type (WT) cells. (**C**) Proportion of cells in each cell cycle phase (G0/G1, S, and G2/M), determined by DNA content based on propidium iodide (PI) staining intensity.

Cell cycle distribution of parental EGI-1 and resistant cells was determined by flow cytometry analysis (Figure 6). In both resistant cell lines, we observed a reduction in the proportion of cells in the G0/G1 phase and an increase in the proportion of cells in the G2/M phase, with no change in the proportion of cells in the S phase.

**Figure 7:**
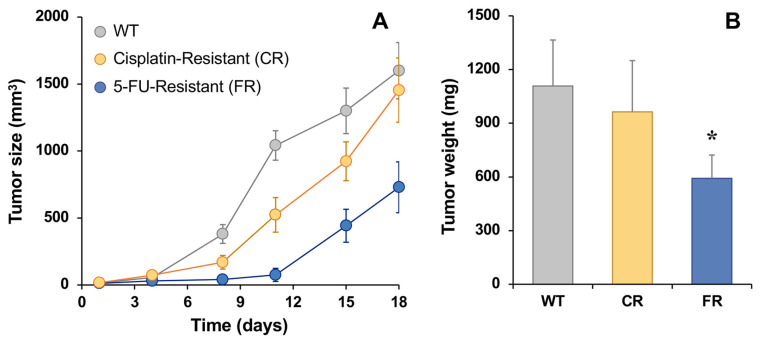
In vivo tumor growth in EGI-1 wild-type (WT), cisplatin-resistant (CR) and 5-fluorouracil-resistant (FR) cells after subcutaneously injecting 10^6^ cells in nude mice (**A**). Tumor weight at the end point (day 18) (**B**). Values are the mean ± SD of four tumors per group. *, *p* < 0.05, comparing tumors formed by implantation of resistant cell lines with those formed by WT cells.

To investigate the tumorigenic capacity of resistant cells, these and WT EGI-1 cells were subcutaneously injected in nude mice, and tumor size was monitored for 18 days since they had a size of ≈25 mm^3^. As shown in Figure 7A, tumor growth was higher in EGI-1 parental cells, with the FR tumor size being the lowest. However, we observed an increase in growth in resistant cells during the last week. Moreover, tumor weight was recorded at the end of the experimental period, which was lower in the FR group than in WT animals, while no significant differences were found between CR and WT tumors (Figure 7B).

### 2.2. Changes in Transportome in Resistant Cholangiocarcinoma Cell Lines

It has been demonstrated that a reduced intracellular concentration of active drugs contributes to the lack of response to anticancer chemotherapy [21]. This can be due to a reduced expression/activity of transport proteins involved in drug uptake (MOC-1A), to an increased expression/activity of proteins able to export drugs (MOC-1B), or to changes in metabolic enzymes that can inactivate drugs or activate prodrugs (MOC-2) [21]. To investigate whether cells resistant to cisplatin or 5-FU presented changes in the expression of transport proteins (transportome) involved in drug uptake and efflux, we compared mRNA levels in resistant and WT EGI-1 cells using TLDAs.

**Figure 8:**
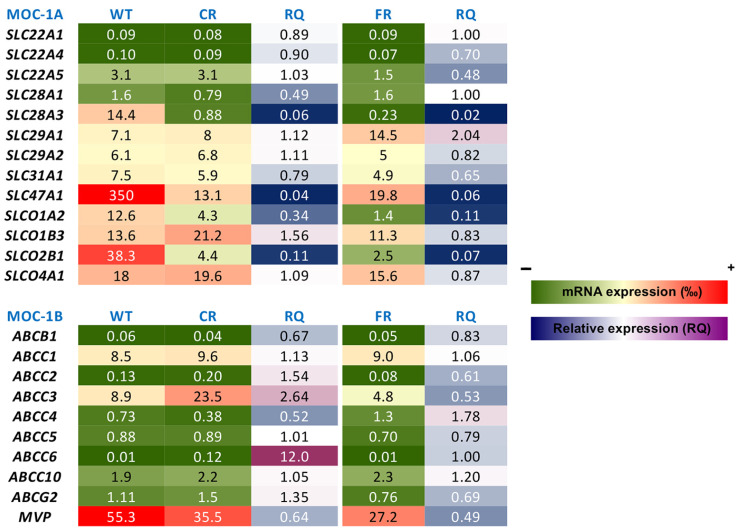
Heatmap of mRNA expression levels of genes involved in mechanisms of chemoresistance (MOC) in cisplatin-resistant EGI-1 cells (CR), 5-fluorouracil-resistant EGI-1 cells (FR), and the parental wild-type (WT) cell line. Expression levels were determined by RT-qPCR using TaqMan Low-Density Arrays (TLDAs). Values are the mean of 9 measurements from 3 separate cultures. For each gene, the value of 2^−ΔCt^ was calculated, where ΔCt represents the difference between Ct of the target gene and the mean Ct of normalizing genes (*GAPDH* and *ACTB*). Data are expressed as parts per thousand (‰) relative to the expression of the normalizing genes. RQ represents the ratio between the expression levels of resistant cells and WT cells. In the color scale bars, colors from green to red correspond to low or high mRNA levels and blue to magenta show a drop or elevation in mRNA levels in resistant cells compared with parental EGI-1 cells.

As shown in Figure 8, in MOC-1A the most significant changes associated with chemoresistance in both CR and FR cells were a substantial reduction in the levels of *SLC28A3*, which encodes the concentrative nucleoside transporter 3 (CNT3), and a decrease in the expression of *SLC47A1* and the organic anion transporters *SLCO1A2* and *SLCO2B1*, which encode MATE1, OATP1A2, and OATP2B1, respectively. Regarding MOC-1B (Figure 8), we found overexpression of *ABCC3*, which encodes multidrug resistance-associated protein 3 (MRP3), only in cells resistant to cisplatin, and higher levels of *ABCC4*, which encodes MRP4, in cells resistant to 5-FU. In CR cells, we also observed an increase in *ABCC6* (MRP6) expression levels compared to WT parental cells (RQ = 8.2); however, despite this relative upregulation, the overall expression remained very low, likely insufficient to reach functionally relevant levels.

To investigate whether subcutaneously grown tumors in vivo retain the gene expression changes associated with chemoresistance (despite the absence of continued drug exposure) we analyzed the expression of the key resistance-related transporters in tumors derived from WT and resistant cell lines (Supplementary Figure S1). The most relevant change was that *SLC28A3* expression was fully restored in both CR and FR tumors.

### 2.3. Cytostatic Effect of Other Drugs in Resistant Cholangiocarcinoma Cell Lines

Next, we wanted to investigate whether in cells resistant to cisplatin or 5-FU there was cross-resistance to other antitumor drugs used as a second line or in patients with specific characteristics to treat this type of biliary cancer. The platinum-containing bile acid derivative Bamet-UD2 was also included among the compounds analyzed. Table 1 shows the IC_50_ values obtained in EGI-1 WT cells and in the two resistant sublines.

CR cells were more resistant to 5-FU and, to a lesser extent, to platinum derivatives like cisplatin, oxaliplatin, and Bamet-UD2 than WT cells, while FR cells were significantly more resistant to irinotecan and gemcitabine than WT cells, and they were slightly less sensitive to paclitaxel, but no cross-resistance to platinum derivatives was found.

## 3. Discussion

The establishment of cisplatin- and 5-FU-resistant EGI-1 CCA cell lines through prolonged exposure to escalating drug concentrations over nine months successfully generated models exhibiting significant chemoresistance. The observed increase in IC_50_ values of over three-fold for both resistant cells compared to the WT parental cells confirms the acquisition of resistance, aligning with previous studies that have demonstrated similar resistance development in CCA cell lines to other drugs [19,20].

Phenotypically, both CR and FR cells displayed reduced proliferation rates, increased doubling times, and diminished migratory and colony-formation abilities. These alterations suggest a trade-off between drug resistance and proliferative capacity, a phenomenon observed in several cancer models where resistance acquisition is accompanied by a slower growth phenotype [22,23]. The observed discrepancy between general proliferation assays and the diminished colony-forming capacity of resistant cells may reflect the distinct cellular behaviors each assay captures. While standard proliferation assays measure overall population growth over time, colony-formation assays evaluate the ability of individual cells to survive, adhere, and expand clonally; processes that may be affected by alterations in adhesion, survival pathways, or cellular plasticity. The impaired colony-forming ability of resistant cells, despite their sustained growth under standard conditions, may indicate a loss of clonal self-renewal or heightened vulnerability to stress associated with seeding [24].

Cell cycle analysis revealed a shift in resistant cells, with a decreased proportion in the G0/G1 phase and an accumulation in the G2/M phase, while the S phase remained unchanged. This redistribution may reflect a cell cycle arrest at the G2/M checkpoint, potentially due to the activation of the DNA damage response or alterations in cell cycle regulators, contributing to the observed slowdown in proliferation. Similar results of cell accumulation in the G2/M phase of the cell cycle have been found in cisplatin-resistant non-small cell lung cancer cells [25] or carboplatin-resistant ovarian cancer cells [26].

Our in vivo experiments demonstrated that both CR and FR cells retained their tumorigenic potential after subcutaneous injection into nude mice, exhibiting slow tumor growth rates in the first few days, but with comparable or even higher growth rates than WT cells at later times. These results suggest that, despite their reduced proliferative and migratory capacity in vitro, resistant cells retain the ability to form tumors in vivo. The increased proliferative capacity of the resistant cells in vivo, evident from a few days after subcutaneous implantation in animals, compared to their slow growth in vitro, can be attributed to the fact that, in this model, the pharmacological pressure was removed. It has been previously described that resistant tumor cells can enter a state of dormancy in response to therapies and subsequently reactivate when conditions are favorable, such as the absence of drugs, and that dormant cancer cells can play a crucial role in tumor relapse [27]. The fact that *SLC28A3* expression was fully restored in both CR and FR tumors could result in an increased nucleoside-uptake capacity, which could partly explain the accelerated proliferation observed once drug pressure was withdrawn, as concentrative transport of nucleosides supplies the nucleotide pool required for rapid DNA replication.

The biological features and molecular mechanisms of these drug-withdrawn cells, which regain proliferative capacity in the absence of drug pressure, are currently being investigated in depth in a follow-up study.

At the molecular level, TLDA-based transportome profiling revealed marked downregulation of *SLC28A3* (CNT3), *SLC47A1* (MATE1), *SLCO1A2* (OATP1A2), and *SLCO2B1* (OATP2B1) in cisplatin- and 5-FU-resistant cell lines. The reduced expression of CNT3, which mediates the cellular uptake of nucleoside analogues [28], may contribute to the reduced intracellular accumulation of 5-FU and explain the tendency for reduced sensitivity to gemcitabine. Although MATE1 has been implicated in the extrusion of oxaliplatin [29], the downregulation observed in MATE1 expression here is unlikely to be directly responsible for the acquired resistance to this drug. None of the changes found in MOC-1A can explain cisplatin resistance, as this drug is primarily taken up by the high-affinity copper uptake protein 1 (CTR1, encoded by the *SLC31A1* gene) [16]. Our results showed a moderate reduction in the mRNA expression of this transport protein, but reduced expression of this transporter has been associated with worse response to cisplatin-based chemotherapy in other tumors [30,31]. In addition, the upregulation of *ABCC3* (MRP3) observed in CR cells suggests that an increased efflux activity may further compromise the intracellular retention and efficacy of cisplatin, as we have recently demonstrated a role for this export pump in the efflux of cisplatin, but not of 5-FU and gemcitabine [32]. The slight increase of MRP4 in FR cells could contribute to 5-FU resistance, together with the reduction of CNT3.

The multidrug resistance phenotype observed, particularly in FR cells exhibiting cross-resistance to gemcitabine and irinotecan, underscores the clinical challenge of treating CCA. Resistance to irinotecan is pronounced and does not appear to be mediated by transporter-related mechanisms, suggesting the involvement of alternative pathways, such as reduced expression of carboxylesterase 1 (CES1) and/or increased activity of cytochrome P450 (CYP) or uridine diphosphate glucuronosyltransferase (UGT1A) [21]. Tumors that do not respond to cisplatin are often resistant to other platinum derivatives; however, cross-resistance to other families of drugs is variable. In previous studies, we have shown that bile acid cytostatic derivatives, such as Bamet-UD2, are effective in hepatobiliary tumors. These drugs have the advantage that they are maintained in the enterohepatic circulation, thus reducing systemic drug levels and, consequently, their toxic effects. They are also taken up by tumor cells, while healthy cells take them up but expel them in the same manner as bile acids [14,15,16]. Although the concentration of Bamets required to have a cytostatic effect is higher than that of cisplatin, the absence of toxicity allows them to be used, and the results obtained in this work suggest that they could be effective in cisplatin-resistant cells. However, the results are more moderate than those obtained in other tumors [15]. It is important to note that the apical sodium-dependent bile acid transporter (ASBT) is the principal transporter responsible for bile acid uptake in cholangiocytes [33]. However, ASBT is not typically associated with mechanisms of chemoresistance. In previous studies, we have observed that ASBT expression remains elevated in a significant proportion of CCAs [16], which supports the rationale for using bile acid-conjugated drugs, such as Bamet-UD2, to enhance drug delivery and efficacy. Nonetheless, the EGI-1 cell line used in our study exhibits negligible expression of ASBT, and this expression remains largely unchanged in resistant cell lines. It is essential to note that various mechanisms may contribute to chemoresistance, including alterations in drug-metabolizing enzymes and the activation of survival pathways, among others [34,35]. However, this work provides a valuable tool for investigating new therapeutic targets to enhance treatment outcomes and overcome chemoresistance in cancers.

## 4. Materials and Methods

### 4.1. Chemicals

For this study, 5-fluorouracil (5-FU), cis-diamineplatinum(II) dichloride (cisplatin), irinotecan hydrochloride, and paclitaxel were obtained from Sigma-Aldrich (Merck, Madrid, Spain). Gemcitabine and oxaliplatin were from Acros Organics (Thermo Fisher, Madrid, Spain). The purity of all these compounds was ≥97%. All other chemicals were of analytical grade. Bamet-UD2 (>90% pure, as determined by thin layer chromatography) was synthesized as previously reported [16].

### 4.2. Cell Cultures

EGI-1 cells, derived from eCCA, were provided by the German Collection of Microorganisms and Cell Cultures (DSMZ, Braunschwaig, Germany) and were cultured in DMEM low glucose (Sigma-Aldrich, Madrid, Spain) supplemented with 10% heat-inactivated fetal bovine serum (FBS), 1% penicillin/streptomycin solution, and 1% non-essential amino acid solution (Gibco, Thermo Fisher, Madrid, Spain).

EGI-1 sublines resistant to cisplatin (CR) or 5-FU (FR) were developed by exposing cells to increasing concentrations of each drug over 9 months, from 2 to 9 µM cisplatin and from 0.5 to 10 µM 5-FU. Resistant cells were maintained in the same culture medium with 20% FBS. Cell morphology, viability, and proliferation rates were systematically assessed throughout the development of drug resistance. Unless specified otherwise, resistant cells were continuously cultured in the presence of the drugs. All the cells were periodically tested to confirm that they were mycoplasma-free (MYCOPLASM Gel Form Kit, Biotools B&M Labs, Madrid, Spain).

### 4.3. Cytostatic Effect and Cell Proliferation Rate

Cytostatic effect in vitro was evaluated in EGI-1 cells seeded onto 96-well plates (7500 cells/well for parental cells and 10,000 cells/well for resistant cells). After 24 h, cells were exposed to different concentrations of the test compounds for 72 h, while control cells were maintained with standard culture medium. Cell viability was assessed using the MTT test (Sigma-Aldrich, Madrid, Spain), and the 50% inhibitory concentration (IC_50_) was determined. Proliferation rates were determined by real-time digital holographic microscopy using a HoloMonitor^®^ Live Cell Imaging System (Phase Holographic Imaging PHI AB, Lund, Sweden).

### 4.4. Cell Migration and Colony-Formation Assays

Cell migration was assessed using wound-healing assays, with monolayer wounds imaged at various time points via phase holographic microscopy. Colony-formation assays were conducted by seeding 500 cells per well in 6-well plates and culturing for 7 days. Colonies were fixed in methanol/acetic acid (7:1) at room temperature for 5 min, then stained with 0.05% crystal violet in 25% methanol for 15 min. Excess stain was removed by washing with water. Colonies were subsequently counted, and their area was measured using ImageJ 1.54 software (NIH, Bethesda, MD, USA).

### 4.5. Cell Cycle Assay

Cell cycle distribution was assessed by propidium iodide staining followed by flow cytometry. In brief, cells were collected, washed with cold PBS, and fixed in cold 70% ethanol for at least 30 min. After fixation, cells were washed with PBS, resuspended in a solution containing 20 µg/mL RNase A, and incubated at room temperature for 30 min. Propidium iodide was then added at a final concentration of 20 µg/mL, and flow cytometric analysis was performed using a FACScalibur cytometer (BD, Madrid, Spain), recording at least 50,000 events per sample. Data were analyzed with CellQuest Pro 5.1 software (BD, Franklin Lakes, NJ, USA), and cell cycle phases (G0/G1, S, and G2/M), as well as the proportion of apoptotic cells, were determined from DNA content histograms.

### 4.6. Quantification of Gene Expression

Total RNA was isolated using RNA mini-spin columns with the Illustra RNA Spin Mini RNA Isolation Kit (GE Healthcare, Madrid, Spain). cDNA was synthesized by retrotranscription from total RNA using random primers and reverse transcriptase included in the High-Capacity cDNA Reverse Transcription Kit (Applied Biosystems, Thermo Fisher). Quantitative PCR (qPCR) was conducted using gene-specific primers spanning exon–exon junctions in the target mRNA, as previously described [16], using Taqman Low-Density Arrays (TLDAs) in an ABI Prism 7900HT Sequence Detection System (Applied Biosystems, Waltham, MA, USA). Thermal cycling conditions were the following: single cycles at 50 °C for 2 min and at 95 °C for 10 min, and 40 cycles at 95 °C for 15 s and at 60 °C for 60 s. The mRNA abundance was double-normalized based on *GAPDH* and *ACTB* mRNA abundance.

### 4.7. Tumorigenesis

For in vivo studies, four 8-week-old female mice (Swiss nu/nu), purchased from Charles River Laboratories (Barcelona, Spain), were housed under sterile conditions in a microisolator cage under controlled conditions of temperature (20 °C), humidity, and light/dark cycle (12 h/12 h) in the animal facilities of the University of Salamanca. The animals were fed on standard rodent chow (Panlab, Madrid, Spain) and water ad libitum. All animal procedures were conducted in accordance with institutional guidelines and approved by the Institutional Animal Care and Use Committee (IACUC) of University of Salamanca and Junta de Castilla y Leon (IACUC number 908).

To evaluate the tumorigenic potential of the resistant cell lines, four immunocompromised mice were subcutaneously injected, under anesthesia, with three different cell populations: CR, FR, and WT EGI-1 cells, as control (≈10^6^ cells in 100 µL FBS/PBS, 1:1). In each mouse, cells were injected at three different sites, both flanks and the dorsal region, to allow within-subject comparison. Each site received a distinct cell type. The goal of the experiment was to determine whether the resistant cells were capable of initiating tumor growth in vivo. As such, no randomization or blinding was applied, and no statistical power calculation was performed. Tumor formation was observed at all injection sites in all animals. Tumor size was measured twice weekly, and tumor weight was assessed at the end of the predetermined experimental period, which was selected to ensure inclusion of all experimental animals in the analysis and to avoid reaching humane endpoints due to excessive tumor burden.

### 4.8. Statistical Analyses

The results were analyzed using GraphPad Prism8 software (San Diego, CA, USA). For comparisons between two groups following a normal distribution, Student’s *t*-test for paired or unpaired values was used, as appropriate.

## 5. Conclusions

The development of CR and FR EGI-1 cell lines provides valuable models for studying the mechanisms underlying chemoresistance in CCA. The phenotypic and molecular changes observed underscore the multifaceted nature of resistance, which involves alterations in drug transport, metabolism, and cell cycle regulation. These models can serve as platforms for testing novel therapeutic agents that aim to overcome resistance and improve treatment outcomes for patients with CCA.

## Figures and Tables

**Table 1:** Cytostatic effect of antitumor drugs in wild-type (WT) EGI-1 cells and in cells resistant to cisplatin or 5-fluorouracil (5-FU).

	IC_50_ Value				
Drug	EGI-1 WT	EGI-1 CR	RR	EGI-1 FR	RR
Cisplatin (µM)	12.9 ± 3.6	47.3 ± 3.2 ª	3.7	9.3 ± 1.1	0.7
5-FU (µM)	11.0 ± 3.2	>30 ª	>3	>30 ª	>3
Gemcitabine (µM)	7.5 ± 3.4	12.0 ± 0.3	1.6	14.6 ± 1.5 ª	1.9
Oxaliplatin (µM)	13.9 ± 2.5	24.5 ± 2.1 ª	1.8	20.0 ± 0.1	1.4
Paclitaxel (nM)	7.7 ± 0.8	8.3 ± 1.1	1.1	12.1 ± 1.3	1.6
Irinotecan (µM)	4.5 ± 0.6	5.8 ± 1.1	1.3	44.9 ± 5.8 ª	10
Bamet-UD2 (µM)	163 ± 22	262 ± 39	1.8	205 ± 42	1.4

CR, cisplatin resistant; FR, 5-FU resistant; IC_50_, half maximal inhibitory concentration; RR, resistance ratio; ª, *p* < 0.05, compared with WT cells.

## Data Availability

Data are contained within the article.

## Supplementary Materials

The following supporting information can be downloaded at https://www.mdpi.com/article/10.3390/molecules30143053/s1, Figure S1: mRNA expression of selected genes in tumors.

## Abbreviations

The following abbreviations are used in this manuscript:

5-FU	5-fluorouracil
CCA	cholangiocarcinoma
CR	cisplatin-resistant
FBS	fetal bovine serum
FR	5-fluorouracil-resistant
IC_50_	50% inhibitory concentration
qPCR	quantitative PCR
TLDA	Taqman Low-Density Array
WT	wild-type
