# Supplementary material for: Drug-Resistant Cholangiocarcinoma Cell Lines for Therapeutic Evaluation of Novel Drugs
Source: Molecules. 2025 Jul 21;30(14):3053. doi: 10.3390/molecules30143053 (PMC12299139; doi:10.3390/molecules30143053)
Supplement: Supplementary file 1 [file molecules-30-03053-s001.zip › molecules-3721825-supplementary.pdf]

	EGI cells			Cisplatin resistant cells				5-FU resistant cells			
**MOC-1A**	**WT**	**WT tumor**	**RQ**	**CR**	**RQ**	**CR tumor**	**RQ**	**FR**	**RQ**	**FR tumor**	**RQ**
SLC28A3	14.4	17.8	1.23	0.88	0.06	15.8	1.09	0.23	0.02	15.5	1.07
SLCO1A2	12.6	8.5	0.67	4.3	0.34	5.6	0.44	1.4	0.11	2.9	0.23
**MOC-1B**											
ABCC3	8.9	14.5	1.62	23.5	2.64	23.2	2.60	4.8	0.54	17.4	1.95
ABCC4	0.73	0.66	0.90	0.38	0.52	1.7	2.33	1.3	1.78	1.9	2.60

\- +

mRNA expression (‰)

Relative expression (RQ)

Supplementary Figure S1. Heatmap of mRNA expression levels of selected genes involved in mechanisms of chemoresistance 1 (MOC-1) in cisplatin-resistant EGI-1 cells (CR), 5-fluorouracil-resistant EGI-1 cells (FR) and the parental wild-type (WT) cell line, as well as in tumors formed from these cells. Expression levels were determined by RT-qPCR. Values are the mean of 9 measurements from 3 separate cultures or 8 measurements from 4 tumors per group. For each gene, the value of $2^{-\Delta Ct}$ was calculated, where ΔCt represents the difference between Ct of the target gene and the mean Ct of normalizing genes (*GAPDH* and *ACTB*). Data are expressed as parts per thousand (‰) relative to the expression of the normalizing genes. RQ represents the ratio between the expression levels in resistant cells or in tumors and WT cells. In the color scale bar, colors from green to red correspond to low or high mRNA levels and blue to magenta show drop or elevation of mRNA levels cells compared with parental EGI-1 cells.
